# EP33 Sjögren’s and lymphoproliferative disorders

**DOI:** 10.1093/rap/rkaa052.032

**Published:** 2020-11-03

**Authors:** Khin Yein, Elizabeth Price

**Affiliations:** Great Western Hospital, Swindon, United Kingdom

## Abstract

**Case report - Introduction:**

Sjögren’s syndrome (SS) is a chronic autoimmune inflammatory condition characterised by lymphocytic infiltration of predominantly exocrine glands as well as other organs involving more commonly in skin, lungs, and neurological system. It is associated with increased risk of both benign and malignant lymphoproliferative disorders.

**Case report - Case description:**

A 29-year-old woman with 5-year history of Primary SS (ANA, Ro/La positive) was reviewed in a routine follow up. She has mild dry eyes, intermittent vasculitic rash around the ankles, occasional pleuritic chest pain but no cough or dyspnoea initially. She used artificial tears but took no regular mediations.

Oxygen saturation at rest was 99% and BP 127/70. Heart sounds were normal, and chest was clear.

Blood results showed alanine transaminase (ALT) had risen from 50 (normal 5-45) to 145 over 6 months. Viral screen (hepatitis, HIV, CMV, EBV) and liver ANA panel were negative. C4 was low 0.09 (n 0.15-0.55), C3 normal and DsDNA antibodies 1 (n 0-15.1). Ig G was high, fluctuating between 27 - 33 (n 7.67-15.9). ESR had been mildly elevated at 26 (n 0-19). CRP had always been within normal limits.

Ultrasound abdomen showed enlarged spleen (15 cm).

CT chest abdomen and pelvis showed multifocal groundless opacification affecting mid to upper zones, bilateral cyst formation, solid nodularity, and mild septal thickening with no pleural effusion. There were multiple prominent bilateral axillary and mediastinal lymph nodes measuring upto15 mm. The spleen measured 15.4 cm.

Six months later, she developed mild dyspnoea on exertion. Examination revealed fine basal crackles. Oxygen saturation remained 99% at rest. TLco was mildly reduced and Kco in lower limit of normal. Echocardiogram was normal.

Ultrasound guided core biopsy of axillary lymph node showed benign reactive features with follicular hyperplasia and plasmacytosis.

The diagnosis of lymphocytic interstitial pneumonitis was made. She was started on a reducing dose of Prednisolone 30 mg and Mycophenolate stepwise increment to 1 gm BD. Chest pain resolved shortly after starting glucocorticoid therapy and exercise tolerance improved.

Liver enzyme normalised.

**Case report - Discussion:**

The diagnosis of lymphocytic interstitial pneumonia (LIP) in this patient was incidental from the retrospective investigation of the persistently abnormal unexplained liver function test. Splenomegaly is a recognised manifestation of active systemic SS. The main differential diagnoses are lymphoma and other types of interstitial lung diseases (ILD).

Her presentation was insidious and there were no constitutional symptoms. Her ANA, Ro, La and Rheumatoid factor were strongly positive. She has hypergammaglobulinemia which is commonly associated with LIP.

Histological findings were typical reactive changes with characteristic polymorphic plasmacytosis. In the absence of malignant lymphoid cells and radiological features of ground glass changes and bilateral cysts formation suggested that the most likely diagnosis was LIP secondary to primary SS.

The management strategies vary for different types of ILD based on their etiological conditions.

There are no specific guidelines for the treatment of LIP. For initial acute stage, glucocorticoid therapy is usually prescribed for symptomatic improvement. For later stages and cysts, immunosuppressants are used and depending on the severity, azathioprine, cyclosporin, rituximab and cyclophosphamide have been used.

Median survival for LIP is 11.2 years. Increased risk of secondary infection because of immunosuppressive therapy is common and there are recommendations for atypical chest infection prophylaxis.

**Case report - Key learning points:**

Interstitial lung disease is commonly associated with SS and predicts a higher mortality rate.

Glucocorticoid is the main stay of treatment for ILD acute stage with active inflammatory changes.

For refractory cases and chronic cystic stages on radiological findings, further immunosuppression therapy may be required.

Standardised treatment guidelines are not available.

Secondary chest infections may be seen in those with cystic disease and on immunosuppression.

Close monitoring and MDT approach is required in the management of LIP.

LIP may transform into lymphoma or co-present with lymphoma associated with SS.

